# The economic efficiency of aid targeting^☆^

**DOI:** 10.1016/j.worlddev.2022.106062

**Published:** 2022-12

**Authors:** Ariel BenYishay, Matthew DiLorenzo, Carrie Dolan

**Affiliations:** a William & Mary, United States; b Old Dominion University, United States

**Keywords:** Foreign aid, Targeting, Subnational

## Abstract

How efficient is the targeting of foreign aid to populations in need? A long literature has focused on the impacts of foreign aid, but much rarer are studies that examine how such aid is allocated within countries. We examine the extent to which donors efficiently respond to exogenous budget shocks by shifting resources toward needier districts within a given country, as predicted by theory. We use recently geocoded data on the World Bank’s aid in 23 countries that crossed the lower-middle income threshold between 1995 and 2010 and thus experienced sharp aid reductions. We measure locations’ need along a number of dimensions, including nighttime lights emissions, population density, conflict exposure, and child mortality. We find little evidence that aid project siting is increasingly concentrated in worse-off areas as budgets shrink; the only exception appears to be a growing share of funding in more conflict-affected areas. We further analyze the relationship of health aid to child mortality measures in six key countries, again finding little evidence of efficient responses to budget shocks. Taken together, these results suggest that large efficiency gains may be possible in the distribution of aid from the World Bank and other donors.

## Highlights

• We assess how efficiently foreign aid is targeted within countries to populations in need.
• We use recently geocoded data on World Bank aid in 23 countries that crossed the lower-middle income threshold between 1995 and 2010 and experienced sharp aid reductions.
• We measure locations’ need along many dimensions, including nighttime lights emissions, population density, conflict exposure, and child mortality.
• We find little evidence that aid project siting is increasingly concentrated in worse-off areas as budgets shrink; the only exception appears to be a growing share of funding in more conflict-affected areas.
• We further analyze the relationship of health aid to child mortality measures in six key countries, again finding little evidence of efficient responses to budget shocks.

## 1 Introduction

How well directed are foreign aid resources toward the needs of the populations they aim to benefit? In particular, how efficiently are foreign aid organizations allocating resources *within* developing countries? Research over the past several decades has primarily explored the degree to which donors, particularly bilateral donors, allocate aid to specific countries based on various political and economic factors (e.g., colonial ties, political alignment in international institutions, trade ties). However, little is known about the degree to which donors succeed in directing development resources within each country toward different locations in ways that maximize welfare. Recent work has shown that, at least in the cross-section, aid allocations are *positively* correlated with wealth (Öhler, Negre, Smets, Massari, & Bogetić,2019; Öhler et al., 2019) and child health (Kotsadam, Østby, Rustad, Tollefsen, & Urdal, 2018), and that aid project allocations sometimes – but not always – respond to political and ethnic motives (Jablonski, 2014, Brazys et al., 2017, Dreher et al., 2019). Yet determining how efficiently the vast majority of aid is allocated remains an open question.

We develop a simple theoretical model of efficient aid allocation – where “efficiency” is defined as an allocation that maximizes overall welfare – across districts within a recipient country. The model predicts that if donors allocate aid efficiently, the share of aid going to needier areas should increase when the overall budget available for the country shrinks. We draw on these insights to derive empirical tests of allocative efficiency, accounting for potential differences in costs of delivering aid across areas that exhibit higher or lower need. We do this by using geo-referenced data on the activities of the World Bank (WB) and measures of underlying conditions at both the district- and region-level within developing countries. A key challenge in empirically assessing the efficiency of aid allocation is that the characteristics of each country’s regions are not randomly assigned. Long-term causes of differences in characteristics are likely to be correlated with omitted factors (such as institutional quality, e.g., Michalopoulos & Papaioannou, 2013), while short-term causes elicit a narrow set of donor responses (such as emergency aid in response to disasters or violence, e.g., Bezerra & Paul, 2016).

To overcome these challenges, we exploit exogenous variation in the total amount of aid that the WB can provide to each country, recently detailed by Galiani, Knack, Lixin Colin, and Zou (2017). One of the largest and most sophisticated donors, the World Bank has provided more than $1.6 trillion in aid since 1947, following exacting processes of project preparation, review, and implementation. We compare differences in aid allocations across high and low-need areas under varying total aid budgets. We find little evidence of changes driven by aid shocks. This is true for a variety of measures of aid, as well as for a range of subnational need indicators, including nighttime lights, population density, remoteness, and a composite measure generated via principal components analysis that incorporates these proxies along with a variety of other features. The only exception we identify is a substantial increase in project siting and funding in districts where baseline conflict fatalities were highest. In a subset of countries, we assess whether health aid projects are more likely to be located in areas with initial higher child mortality after the Bank’s funding shrinks, again finding no such evidence. We check that differences in project implementation costs across more or less needy areas (which could mute any response to differences in need) also do not materialize. Other robustness checks bear out our primary findings.

We conclude that the World Bank – one of the leading aid institutions – does not appear to direct aid resources within countries towards the areas with the greatest need. These findings suggest that the Bank’s subnational targeting diverges from the organization’s stated goals. We cannot rule out that the Bank’s targeting does efficiently reflect other, implicit aims (for example, by extracting key national-level policy reforms from domestic governments in exchange for politically motivated aid allocations to less poor areas). Nonetheless, in showing the degree to which aid flows primarily to less poor subnational areas, our results suggest the implicit gains from these trade-offs would have to be very large to be make the overall distribution of benefits pro-poor.

Our paper is organized as follows: in Section 2, we discuss the related literature, and in Section 3 we provide a conceptual framework and generate testable predictions. In Section 4, we describe our data and lay out our empirical methodology, presenting results in Section 5. We provide robustness checks in Section 6. In Section 7, we focus our scope more narrowly on health aid and need measures. Finally, we consider whether there are offsetting geographic differences in costs in Section 8, before offering conclusions in Section 9.

## 2 Literature review

A large literature has examined the impacts of aid on a wide variety of economic, political, and social variables at the cross-national and, increasingly, at the subnational level (e.g., Crost et al., 2014, Rajan and Subramanian, 2008, Rajan and Subramanian, 2011, Clemens et al., 2012, Galiani et al., 2017, Marty et al., 2017, Bazzi et al., 2012, Dreher et al., 2019, Isaksson and Kotsadam, 2018, Isaksson and Kotsadam, 2018, Knutsen and Kotsadam, 2020). Our work builds on the much scarcer but growing literature examining how aid resources are allocated within countries. A related literature concentrates on the efficiency of what might be considered micro-targeting, i.e., allocations to households within villages (c.f., Alatas et al., 2012, Baird et al., 2013). Our paper complements these literatures by exploring the efficiency of allocations at the meso-scale (i.e., between macro- or country-scale allocations and micro- or household-scale allocations). Briggs (2017) explores the cross-sectional relationship between wealth (measured in household surveys) and aid projects funded by the WB and African Development Bank (ADB), finding that wealthier areas are disproportionately funded. Briggs (2018a) reinforces this finding using more granular units of analysis, and Briggs (2018b) extends the analysis to a wide variety of donors and finds that aid projects tend to be co-located with the rich in Nigeria, Senegal, and Uganda.^1^ Similarly, Öhler et al. (2019) find little consistent evidence that WB aid is targeted toward subnational regions with higher shares of the population in the bottom 40% of the income distribution. In Nigeria, Kotsadam et al. (2018) find that aid from multiple sources is disproportionately located in areas with initially lower child mortality rates. Nunnenkamp, Öhler, and Andrés (2017) study WB project locations across Indian districts, finding only very weak evidence of any correlation with poverty or other need measures.

Taking a political economy approach, Jablonski (2014) finds that within Kenya, WB and ADB projects are disproportionately located in constituencies with higher incumbent vote shares, while Caldeira (2011) show that government finance in Senegal is allocated disproportionately to swing electoral districts, with little evidence of equity considerations. Song, Brazys, and Vadlamannati (2021) show that the political empowerment of local groups has influenced the allocation of education aid from the World Bank in India. Dreher et al. (2019) show that Chinese foreign aid to African nations is disproportionately provided to a national leader’s home or co-ethnic region, but that WB projects do not exhibit such favoritism. Other studies document similar political biases even in the allocation of humanitarian and emergency aid (e.g., Lio Rosvold, 2020, Eichenauer et al., 2020). Perhaps the only finding indicating pro-poor targeting is from Bendavid (2014), who finds that increases in health aid to a country lead to larger drops in child mortality among the poorest households. There is also evidence of favoritism in the allocation of government grants in Indonesia (Gonschorek, 2021).

These findings comport with the observations of interested stakeholders who have noted that many major aid programs often fail to adequately incorporate equity concerns and, as such, may not reach the most vulnerable and marginalized populations within recipient countries (e.g., Chi, Bulage, & Østby, 2019). Studies that do find that aid responds to need are limited to specific sectors (Bendavid, 2014). However, many of the above-mentioned studies focus on only one or a handful of countries (e.g., Caldeira, 2011, Jablonski, 2014, Kotsadam et al., 2018, Nunnenkamp et al., 2017) or are limited to Africa (Briggs, 2017, Dreher et al., 2019). More recent work has helped remedy this problem by expanding the spatial domain under consideration (e.g., Öhler et al., 2019, Briggs, 2021). Indeed, while recent studies have expanded the list of countries included in their analyses, the temporal domains of these studies remain limited. For instance, Öhler et al. (2019) focus on the 2004–2014 period but aggregate their measures into a cross-sectional regional analysis. Briggs (2021) studies the 1995–2005 period, though uses only nighttime light luminosity as a proxy for local wealth, which (Öhler et al.2 (2019)) point out is a noisy measure for poverty. In sum, though existing findings are somewhat mixed, the preponderance of evidence appears to suggest that subnational aid allocation tends not to respond to various metrics of need within countries or that it is driven by political factors.

Our goal is to build on existing studies in a few ways. First, we expand on the scope of existing work in terms of data coverage. The study most similar to ours is Briggs (2021), which uses nighttime luminosity as a proxy for local need. We significantly expand the set of measures of that serve as proxies for local need in the analysis below. Second, we adopt an approach recently developed in the cross-national aid allocation literature that attempts leverage exogenous variation in aid budgets to draw better inferences about the relationship between need and allocation given an aid shock. Specifically, we examine whether a variety of potential proxies for local need become stronger or weaker predictors of aid allocation when a country becomes ineligible for receiving IDA assistance, following Galiani et al. (2017). Before outlining that approach, however, we develop a simple model of the aid allocation process to derive clear predictions.

## 3 Conceptual framework

### 3.1 Motivating model

Like Collier and Paul (2001) and Collier and Paul (2002), we begin with a model of optimal aid allocation for the sake of developing a baseline against which we can compare actual allocation patterns. Our goal is to link our understanding of aid efficiency to the workhorse choice models and to motivate the empirical analysis (rather than making a theory contribution). Thus, we consider a simple model of public resource allocation across two different regions with varying levels of underlying need. A development agency or social planner aims to maximize the welfare in these regions, subject to a traditional budget constraint:

$$u(a_{1},\;x_{1})+u(a_{2},x_{2})$$

s.t.

$$B=a_{1}\;*\;c\left(x_{1}\right)+a_{2}\;*\;c(x_{2})$$

Where

$a_{i}$: allocation of public services to area i.

$x_{1}$: conditions in area i, with higher values indicating better conditions.

u(.) is utility in area i, $\partial u/\partial a>0,\partial u/\partial x>0,\partial^{2}u/\partial a^{2}<0$, and $\partial^{2}u/\partial x^{2}<0$.

*B*: Total budget available. c(.): unit cost of services, which may vary by conditions in area i (based on an implicit production function)

For our example, say that region 2 is initially worse off than region 1 ($x_{2}<x_{1}$).

In general, one can show that if the budget constraint is exactly satisfied, donors will allocate public services so that the ratio of marginal utilities across the two regions exactly equals the ratio of marginal costs:

$$\frac{\frac{\partial u\left(a_{1},\;x_{1}\right)}{\partial a_{1}}}{\frac{\partial u\left(a_{2},\;x_{2}\right)}{\partial a_{2}}}=\;\frac{c(x_{1})}{c(x_{2})}$$

To make progress on the responsiveness of the ratio of marginal utilities to shifting budgets, we need to make some assumptions on the functional form of the utility function (specifically, on the separability of public services and conditions). Say that public services and conditions are substitutes, so that donor and government efforts can (at least partially) offset deficient conditions. For our purposes, say that

$$u\left(a,x\right)={\left(a+\mathrm{bx}\right)}^{\rho }$$

where *b* and ρ are constants that shape the substitutability in *a* and *x* and curvature of the utility function. One could introduce additional non-linearity in substitutability of *a* and *x*, which could magnify or dampen responses. We otherwise maintain the same assumptions as our general setup, and thus for (∂u/∂x>0) we require b>0, and for $\partial^{2}u/\partial a^{2}<0$, and $\partial^{2}u/\partial x^{2}<0$, we require ρ<1.^2^

We can then pin down interior solutions^3^ for optimal public services $a_{1}^{*}$ and $a_{2}^{*}$ as a function of initial conditions and the overall budget:

$$a_{1}^{*}={\left(1+\frac{\gamma c\left(x_{1}\right)}{c\left(x_{2}\right)}\right)}^{-1}b\left(\gamma x_{2}-x_{1}\right)+\frac{\gamma B}{c\left(x_{2}\right)}$$

and

$$a_{2}^{*}={\left(1+\frac{\gamma c\left(x_{2}\right)}{c\left(x_{1}\right)}\right)}^{-1}b\left(\gamma x_{1}-x_{2}\right)+\frac{\gamma B}{c\left(x_{1}\right)}$$

where $\gamma ={\frac{c\left(x_{1}\right)}{c\left(x_{2}\right)}}^{\frac{1}{\rho -1}}$.

We can then show that, if the marginal costs of public services are different across the two regions (such that $c(x_{1})\;\neq \;c(x_{2})$), the difference between $a_{1}^{*}$ and $a_{2}^{*}$ will change as the total budget constraint changes:

$$\frac{\partial (a_{2}^{*}-a_{1}^{*})}{\partial B}\;\neq \;0$$

The direction of the response will be ambiguous and depend on the ratio of costs (i.e., whether $\frac{c(x_{1})}{c(x_{2})}$ is greater than or less than 1). As we discuss in the ensuing sections, there are good reasons to believe either is true (i.e., that $\frac{c(x_{1})}{c(x_{2})}$ is either greater than or less than 1). We thus generate the following testable prediction:($H_{1}$) The difference in the levels of aid funding provided to regions with worse conditions and those with better conditions should grow or shrink when total aid for the country shrinks, depending on the ratio of marginal costs across the regions

Here, we also consider the special case in which $c(x_{1})=c(x_{2})$. As we show below, the difference between $a_{2}^{*}$ and $a_{1}^{*}$ will no longer respond to changes in the total budget. We therefore instead consider how the ratio of $a_{2}^{*}$ and $a_{1}^{*}$ responds to the total budget, thus again allowing us to generate testable predictions.

In this setting of equal marginal costs across the two regions,

$$a_{2}^{*}-a_{1}^{*}=b\left(\gamma x_{1}-x_{2}\right)>0$$

and the differences in the levels of public services going to the regions are constant, even as total budget rise and fall. Simply put, the public services simply compensate for any differences in initial conditions, magnified or dampened by the constant b. However, in this situation, we can still generate testable predictions a the ratios of the services going to these regions will not remain constant when budgets fall, as

$$\frac{a_{2}^{*}}{a_{1}^{*}}=1+\frac{2b\left(\gamma x_{1}-x_{2}\right)}{b\left(\gamma x_{2}-x_{1}\right)+\frac{B}{c\left(x_{2}\right)}}$$

and thus the response of this ratio to changes in B will be

$$\frac{\partial \frac{a_{2}^{*}}{a_{1}^{*}}}{\partial B}=2b\left(\gamma x_{1}-x_{2}\right)\left(-1\right)b\left(\gamma x_{2}-x_{1}\right)+{\frac{B}{c\left(x_{2}\right)}}^{-2}\left(\frac{1}{c\left(x_{2}\right)}\right)<0$$

That is, even in the case where marginal costs are equal across regions, the share of resources being devoted to regions with worse conditions should increase as a donor’s overall budget in a country shrinks. Importantly, we have only assumed that public services and conditions are additively separable substitutes.

We thus reach a second testable prediction:($H_{2}$) The ratio of aid funding provided to regions with worse conditions relative to those with better conditions should grow (or shrink) when total aid for the country shrinks (or grows), even if marginal costs are equal across regions

These predictions comport with statements by the World Bank illustrating its logic in shifting funding priorities as countries transition out of IDA eligibility. For example, during Kenya’s transition out of IDA eligibility in 2014, the Bank made clear in its Country Partnership Strategy that “[a]nother key to help target support for the poor is to focus on agriculture, a high priority since it has such a direct link with helping families in rural areas where a majority of Kenyans live” (World Bank, 2014a, vi). As such, this new Country Partnership Strategy for Kenya reflects an intention to efficiently allocate resources under a situation of changing lending eligibility conditions. Importantly, since a key part of the World Bank’s approach involves consulting with recipient countries in formulating Country Assistance Strategies, there is evidence that recipient governments take into consideration shifting budgets in designing poverty reduction strategies. For example, the 2003 Country Assistance Strategy for Macedonia noted that the government decide to “reduce the scope of work” in its Poverty Reduction Strategy Paper once Macedonia became ineligible for IDA assistance (World Bank, 2003, 5, footNote 5). The development goals articulated in what become the Country Assistance Strategy emphasized both “the efficient management of public resources” and the need to “protect the most vulnerable” (World Bank, 2003, 20).

### 3.2 Alternative assumptions

Our conceptual framework makes a number of important assumptions that lead to our predictions. Firstly, we assume b>0, making conditions and aid allocations substitutes (rather than complements). One reason this could fail to hold would be if aid impacts are correlated negatively with need and are actually negative in higher-need areas. However, there is actually little evidence that project impacts are in fact worse in higher need areas. At the cross-country scale, Denizer, Kaufmann, and Kraay (2013) document that country governance and economic growth dynamics explain very little of the variation in World Bank project outcomes. Project results do vary substantially within countries, as Denizer et al. (2013) show, but not primarily on the basis of regional characteristics. At the same time, a spate of subnational studies in the health sector suggest that health interventions may actually be more effective in higher need areas. Whittington, Jeuland, Barker, and Yuen (2012) and Benjamin-Chung and Colford (2016) both conduct meta-analyses of common health interventions and find that their cost effectiveness depend critically on herd protection dynamics, meaning that impacts are greater in higher need areas compared to lower need areas with more existing coverage. Even absent herd protection, a variety of health interventions appear to have disproportionate impacts on people with worse initial conditions. For example, Thomas et al. (2003) show that iron supplementation and deworming has particularly large impacts on those whose baseline hemoglobin levels were particularly low.

The only paper showing greater impacts in lower need locations comes from Europe, where Becker et al. (2013) study the impacts of fiscal transfers to lower income regions and find that, among these regions, only those with sufficient human capital and good enough institutions are able to turn transfers into faster growth in incomes and investment. However, even in this context, the authors do not find negative impacts of fiscal transfers in lower income regions. Moreover, the nature of these fiscal transfers in the European context – direct governmental transfers to subnational administrative units – are quite different than many foreign aid projects. Taken together, we argue there is little consistent evidence that indicates that conditions and aid allocations are complementary in terms of utility.

A second major aspect of our approach is that it considers the perspective of a single donor rather than multiple donors with potentially competing or complementary objectives, or domestic governments carrying out their own agendas. While a game theoretic approach may be a worthwhile direction for further research, our focus here is on understanding how one major donor determines its own priorities.^4^ Our choice-theoretic focus on one major donor does omit the possibility that other donors or domestic governments respond to the Bank’s allocations by targeting the neediest regions and thus generate aggregate distributions of aid that are pro-poor, but this is unlikely given both the state of donor coordination and the scale of the Bank’s funding. The World Bank Group is the single largest source of official development assistance (ODA), providing over $37 B in 2019 (20% more than the United States, the next largest provider, and 4 times as much as all regional development banks combined, 7 times as much as all United Nations agencies combined, and 10 times as much as the Bill & Melinda Gates Foundation, the largest private donor) (Organization for Economic Cooperation & Development, 2021). Thus, the WB is often playing a leading role in shaping many aspects of aid strategies and activities, including the subnational targeting of these activities.

Moreover, while there have been repeated public commitments to better coordination among donors (including by the WB), there is still very little evidence of improved coordination. These coordination challenges are well documented both at the country level (Chandy and Laurence, 2011, Gore, 2013), but a growing body of evidence is also pointing to the lack of coordination at the subnational level (Öhler, 2013, Findley et al., 2015, Nunnenkamp et al., 2016). Our case study analysis of donor coordination in Nigeria (discussed in Section 9) highlights this phenomenon in one of our sample countries. There is thus little reason to believe that the WB would be acting efficiently by not concentrating resources in the districts with greatest need because it correctly anticipates that other donors and domestic governments will do so in response to its own allocations.

Our approach also relies on a static rather than dynamic objective function. There are multiple channels through which longer term outcomes may flow through to current aid allocations by donors, including donors considering the persistence of poverty in some regions due to their slower growth rates (Wood, 2008). At the subnational region scale, high poverty rates often coincide with the slowest growth rates. This suggests that incorporating dynamic objectives into our model would likely sharpen the response to budget cuts even more in favor of regions with worse poverty, as donors’ allocations would consider not only their currently high poverty but also their slow declines.

Finally, we define donors’ objective function on the basis of joint utility derived from conditions and aid allocations. These comport with the United Nations’ Sustainable Development Goal #1 and the WB’s own “twin goals” of ending extreme poverty (reducing the percentage of people living on less than $1.25/day to less than 3%) and promoting shared prosperity (defined in terms of the living standards of the bottom 40% of the population in every country) (World Bank, 2014b). Thus, we consider poverty and living conditions as particularly salient for our objective function. We recognize that many donors focus on other goals, including human rights, gender equality, environmental conservation, or indeed many of the other Sustainable Development Goals. Our conceptual framework is general enough to admit many of these, to the degree they lead to utility differences across regions on the basis of the conditions in these regions’ and donors’ investments in them. Naturally, there are other objectives that would require different modeling approaches.

## 4 Data and methodology

### 4.1 Foreign aid data

Data on foreign assistance at the subnational level is now available for a growing number of countries via the AidData project at William & Mary. The most comprehensive of these datasets covers the funding provided by the WB two of its arms, the International Development Association (IDA) and the International Bureau for Reconstruction & Development (IBRD). We use the AidData IBRD-IDA Projects Geocoded Research Release Level 1 v1.4.2 (AidData, 2017), which covers projects approved between 1995 and 2014. These projects entailed $630 billion in commitments across Africa, Latin America, Asia and Eastern Europe. The AidData geocoding effort identified 61,243 locations associated with these projects, with the vast majority of these geolocated to at least the district the level.

We structure our analysis data at the level of the second administrative district (typically, the district level). We do so for several reasons: first, the variation of aid at this scale is particularly relevant given how dramatically living conditions vary across districts in most countries, and thus how much of the overall responsiveness of aid to conditions is due to district-level allocations. Second, much of the aid data we rely on is only coded to this scale (i.e., finer geocoordinates are not available). In some cases, this is because the aid projects themselves are intended to have district-wide benefits (such as governance support efforts aimed at the district governments). For robustness, we also repeat our analysis using the first administrative units (typically regions or states).

In each year, we identify whether the district has any newly approved projects, as well as the approximate total committed value of these projects in constant 2011 USD.^5^ We focus on aid commitments rather than disbursements for two reasons: (1) data commitments are much more consistently available than data on disbursements from the World Bank project data underlying the AidData dataset; and (2) aid commitments to each district represent the planned (i.e. targeted) funding, while disbursements may be affected by implementation conditions themselves, thereby creating spurious correlations that reflect implementation rather than targeting. While our first outcome variable (*any projects in the district*) is binary, the other measure (*total committed funds*) is continuous. We normalize the latter measure by baseline population to derive a per capita measure of aid in each district-year. As a robustness check, we also consider the number of locations in each district in which aid projects took place as another outcome, under the logic that more intense project activities are likely to be spread across more locations within a given district. These results are shown in Table 14 in the Appendix.

In our baseline specification, we include aid across all sectors (as the specific choice of sector may well endogenously respond to a location’s characteristics). In Section 7, we narrow our measures to only the health sector. In the Appendix, we also include results narrowed to only the infrastructure sector.

As our Conceptual Framework generates predictions on both the levels and the ratios of aid, we generate measures that reflect each. $a_{\mathrm{it}}$ captures aid to district *i* in year *t*. To account for ratios, we use the natural logs of aid ($\ln(a_{\mathrm{it}}+1)$) for our continuous outcomes measures. As we describe below in our Specifications section, using these measures in our linear empirical specification is akin to estimating effects on the levels and ratios of aid across districts. Table 1.

**Table 1:** Sample countries.

Albania	Dem. Rep. of the Congo	Mongolia
Angola	Djibouti	Nigeria
Azerbaijan	Georgia	Papua New Guinea
Bhutan	Ghana	Solomon Islands
Bolivia	Guyana	Sri Lanka
Bosnia and Herzegovina	Honduras	Ukraine
Cameroon	India	Uzbekistan
China	Indonesia	

To test our theoretical predictions, we use exogenous variation in countries’ overall aid budgets noted and detailed by Galiani et al., 2017. The World Bank uses a classification system to categorize low income countries (LICs) and lower-middle income countries (LMICs), with the threshold first established in 1987 and updated annually based on inflation. Between 1995 and 2010, the period in our study, 23 countries crossed the LMIC threshold.^6^ Once a country cross the income threshold for several consecutive years, it is deemed to be creditworthy and is thus on track to “graduate” from the highly preferential grant funding provided by IDA. In practice, these funding reductions occur two to three years after a country crosses the threshold, as IDA operates under three-year “replenishment periods.” Actual graduation from IDA (complete ineligibility from this funding source) occurs subsequently (its timing is likely endogenous). Galiani et al. show that following crossing the LMIC threshold, countries’ IDA funding as a share of GNI drops by 92%. Importantly, many other donors follow suit, with some even explicitly using the LMIC threshold as a criteria (as in the case of the African and Asian Development Banks). Overall aid budgets as a share of GNI thus shrink by 59% following the crossing (Galiani et al., 2017).

Based on this context, we limit our sample to the 23 countries that crossed the LMIC threshold between 1995 and 2010 (the period over which we have georeferenced aid data such that we can track both pre- and post-crossing allocations). For each country, we identify the year of crossing, as well as the year at which the subsequent IDA replenishment period begins. We construct a dummy variable ${\mathrm{crossed}}_{\mathrm{ct}}$ that indicates whether country *c* at year *t* had crossed the LMIC threshold in the preceding replenishment period. Because overall country budgets drop substantially within three years of threshold crossing, we expect within-country responses to also occur within this timeframe. As a robustness check, we confirm that longer lags do not appear to generate such responses.

### 4.2 Need data

Our study requires data that is available both at high levels of spatial granularity and in a standardized way for many developing countries around the world prior to 1995, when our aid panel begins. In order to identify whether there is any meaningful aid responsiveness to local conditions in a varied and comprehensive way, we obtain a wide array of data derived from a diverse mix of sources. Our primary criteria for inclusion are their availability across the developing world (all of our sample countries), fine spatial granularity (finer than district scale, at a minimum), and time-series availability back to the year 2000 (at a minimum). Our goal is not to test these conditions measures against one another but to identify whether aid targeting is responsive to *any* of them. While the underlying data are available at differing spatial resolutions, we aggregate each of these measures to the district scale using the GeoQuery tool publicly available via AidData. (Goodman, BenYishay, Lv, & Runfola, 2019). GeoQuery overlays district boundary polygons over each of the need datasets and generates means (and other statistics) over all of the grid cells falling into each polygon (with grid cells only partially falling into the polygon weighted by the share of their area within the polygon).

Among our primary set of measures, we use the nighttime lights (NTL) emissions available annually at 1 km grid cells from the DMSP-OLS program. A burgeoning literature documents that the NTL are well correlated with economic activity and other measures of well-being (Dreher and Axel, 2015, Henderson et al., 2017, Bruederle and Anna, 2018). We obtain the mean NTL emissions in 1992 over each district (thus also avoiding known limitations with the NTL data when applied at lower scales). Similarly, we use population totals and density from the CIESIN GPW v3 data, as Briggs (2017) documents the relevance of population density in aid allocations. Again, we aggregate the 2.5 km resolution data to district means in 1995.

Because NTL and population density measures may be particularly correlated with need in urban or peri-urban areas, we also include measures that differentially correlate with well-being in rural areas. Crop productivity and overall vegetation production thus serve as key measures in these situations. We focus on the Normalized Difference Vegetation Index (NDVI) obtained from the NASA Land Long-Term Data Record for 1995 at approximately 4 km grid cells (similar measures have been used at varying scales and from other satellite sources by Burke & Lobell (2017)). This measure captures how “green” a given grid cell appears, with greener cells more productively farmed or forested.

The population, NTL, and NDVI measures all reflect underlying patterns of human land use, i.e., whether a given area is urban, peri-urban, farmland, or other, naturally occurring uses. To more directly incorporate these uses, we rely on the European Space Agency’s Land Cover 2.0.7 product, which uses satellite-based reflectance measures to categorize grid cells at 300 m resolution into 11 categories, including urban, various cropland types (irrigated, rainfed, mosaic), various shrubland, water, etc. We generate measures of the shares of each district that is classified as urban and any type of cropland, thereby indicating the extent of urban or agricultural use.

We further measure the extent of road networks and remoteness of districts using the average travel time to major cities generated by the Joint Research Centre of the European Union. The earliest year for which this data is available is 2000; while not ideal, this data is unlikely to have been affected dramatically by aid allocations between 1995 and 2000. In robustness checks, we confirm that dropping this early period does not alter our results.

A district’s remoteness and access to resources can also reflect long-term drivers of development. We include the district mean distance to the coast and to a major waterway from the Global Self-consistent, Hierarchical, High-resolution Geography Database (GSHHGD), as well as distances to the nearest on-shore petroleum and diamond deposits from the Peace Research Institute Oslo (PRIO) and nearest lootable gold deposit from GOLDATA. Mean distances for districts are determined by disaggregating districts into regular 1x1km grids cells, calculating distances for each cell, and averaging results for each district as implemented by GeoQuery.

A growing literature documents the impacts of governance and institutions on long-term development at the subnational scale. However, standardized measures of these concepts at the subnational scale with coverage across the globe remain quite scarce. As one indicator, we rely on the Uppsala Conflict Data Program Georeferenced Event Database (UCDP-GED) to obtain district means of the number of conflict fatalities occurring between 1990 and 1995.

Finally, we consider childhood mortality data from the Demographic & Health Surveys (DHS) as a particularly important indicator of well-being, albeit one that is only available at a spatially granular level for a small subset of our countries. For Bolivia, Cameroon, Ghana, India, Indonesia, and Nigeria, we are able to generate average under-5 childhood mortality during 1990–1995 for consistent regions over which we can also obtain health aid project locations. While this is a small number of countries, they include large, densely populated countries with many regions and $4.2 billion in WB funding committed during our study time-period.

Summary statistics for our aid and need data are displayed in Table 2.

**Table 2:** Summary Statistics.

	Obs	Mean	SD	Min	Max
New aid projects in district (0/1)	105140	0.074	0.26	0	1
New aid commitments per capita (000’s USD)	105040	7318.9	253709.0	0	46681392
Aid locations per capita (count per person)	104960	5.95	314.0	0	53099.1
Area of district (${\mathrm{km}}^{2}$)	105140	0.44	1.65	0.00000100	49.6
Total population (millions of people)	105060	0.50	1.22	0	29.5
Population density (people per ${\mathrm{km}}^{2}$)	105080	340.7	1184.9	0.24	42914.8
Nighttime lights (index value)	105140	4.76	9.87	0	63
NDVI (index value)	105140	4601.4	1304.5	675.0	7631.1
Urban share (%)	105120	0.027	0.11	0	1
Cropland share (%)	105120	0.48	0.34	0	1
Road dist. to cities (minutes of travel time)	104960	336.8	447.6	3.14	9467.8
Dist. to coast (meters)	105140	417.9	507.4	0	2665.7
Dist. to waterway (meters)	105140	43.6	44.6	0	457.9
Dist. to petroleum (meters)	105140	270.2	331.9	0	2277.8
Dist. to gold (meters)	105140	333.1	228.3	3.40	1619.5
Dist. to diamonds (meters)	105140	626.8	532.7	4.41	2336.2
Conflict fatalities (annual fatalities in district)	103620	0.012	0.20	0	10.0

### 4.3 Specification

Our first theoretical prediction ($H_{1}$) states that the difference in the levels of aid funding provided to regions with differing conditions should change in response to changes in total aid flows a given country. When implementing this empirically using many regions, we assess how the levels of aid in each region vary with their conditions heterogeneously with respect to shocks in total aid flows. That is, we estimate the following specification:

$$a_{\mathrm{ict}}=\beta_{0}+\beta_{1}{\mathrm{Crossed}}_{\mathrm{ct}}+\beta_{2}{\mathrm{Conditions}}_{\mathrm{ic}}+\beta_{3}{\mathrm{Crossed}}_{\mathrm{ct}}\;*\;{\mathrm{Conditions}}_{\mathrm{ic}}+D_{c}+D_{t}+{∊}_{\mathrm{ict}}$$

where ${\mathrm{Conditions}}_{\mathrm{ic}}$ are the need measures described in our Data section and $D_{c}$ are country fixed effects absorbing all time-invariant characteristics common to all districts in each country and $D_{t}$ are year fixed effects absorbing the effects of all common temporal shocks. In some cases, higher values of ${\mathrm{Conditions}}_{\mathrm{ic}}$ reflect better conditions (i.e. NTL) while in others, they reflect worse (i.e. conflict) or non-ranked outcomes (i.e. NDVI). We estimate this specification using ordinary least squares and use two-way clustering of standard errors by country and by year following Cameron, Gelbach, and Miller (2011). Multi-way clustering extends the insights offered by Bertrand, Duflo, and Mullainathan (2004) to simultaneously cover clustering along multiple dimensions (separately), thereby typically providing more conservative variance estimates (i.e., those that are less likely to reject the null hypothesis). For our binary outcome measures, this specification takes the form of a linear probability model.

Our coefficient of interest is $\beta_{3}$, reflecting the change in allocations to higher-need districts after a country crosses over the LMIC threshold. The linear model with interactions between region conditions and country-level shocks in aid is equivalent to testing how the differences in aid between region pairs vary in response to the differences in their conditions and the total country aid budget. This difference-in-differences specification thus combines cross-sectional variation in need and temporal variation based on country threshold crossing. Again, Galiani et al. (2017) show how the threshold crossing generates sharp drops in aid, even after controlling for smoother trends in a country’s aid and economic trajectories. Thus, the temporal shock at the country-level can be considered exogenous from the perspective of regions within that country, while our use of baseline conditions makes their interaction with the threshold crossing unconfounded with other time-invariant features. Our coefficient of interest essentially identifies the within-country heterogeneity in the response to the temporal shock across districts’ varying need conditions. The large overall country-level drop in aid relative to GNI of 59% identified by Galiani et al. (2017) coupled with dramatic variation in need across districts within each country together provide substantial scope for us to observe the district-level heterogeneity in aid responses.

We similarly test our second hypothesis ($H_{2}$) via the same specification, but instead use $\ln(a_{\mathrm{it}}+1)$ as our outcome measure. Because the differences in these logs of aid across regions are equal to the ratios of the aid, this specification is equivalent to testing whether the ratios of aid flows to region pairs change with respect to the total country-level budget. We can conduct this test for our continuous measure (*total committed funds*).

Because we consider multiple outcome variables and 14 different conditions variables, addressing multiple comparisons issues is crucial. We do so in several ways. First, we use principal components analysis to generate three orthogonal summary measures of conditions. Factor loadings are shown in Table 3. The first component essentially reflects urbanization, most heavily weighting nighttime lights, population density, urban share, and road distance travel to urban centers. The second and third factors reflect a broader set of conditions from the remaining variables.^7^ We use these components to test whether the results on individual variables are statistically similar to those derived from these summary measures. Additionally, we consider whether the results for individual variables are both logically and statistically consistent across our multiple outcome measures. Taken together, these approaches allow us to guard against over-rejection of our hypotheses based on a small number of potentially randomly occurring correlations in the data.

**Table 3:** Principal Components.

	Comp 1	Comp 2	Comp 3
Area of district	−.1742082	.30714	.0661096
Total population	.1042281	.1023339	−.3059528
Population density	.4414385	.2118376	.2158132
Nighttime lights	.50863	.2051469	.1490906
NDVI	.0185262	−.5414113	.2754869
Urban share	.478758	.2338979	.282047
Cropland share	.1913811	−.115942	−.5953942
Road dist. to cities	−.3263772	.2148625	.3643931
Dist. to coast	−.221136	.5007026	−.0996497
Dist. to waterway	−.2022248	.3269403	−.0424721
Dist. to petroleum	−.1603379	.034189	.1463227
Dist. to gold	.0483062	.1890697	−.2446885
Dist. to diamonds	−.1027704	−.0789778	.3111252
Conflict fatalities	.0641467	.0065333	.026141

## 5 Results

We begin by examining the changes in whether any IDA or IBRD project is located in a given district. In Table 4, we show coefficients on each condition variable, with these variables labeled in column headings.^8^ We observe that, before crossing the LMIC threshold, projects are more likely to be located in districts with larger, more dense populations. Not surprisingly, these districts also emit more NTL, have higher shares of areas classified as urban, have shorter average travel times to urban centers, and are closer to coasts. This set of correlations is consistent with those identified in Briggs (2017). We do not detect statistical correlations with our other conditions variables, including greenness and conflict fatalities. Our principal components exhibit a similar pattern: component 1, which places substantial weight on population, NTL, and urban status, shows a strong correlation with whether a project is sited in a district, while the other components do not.

**Table 4:** Any new projects in district.

**Panel A**								
	(1)	(2)	(3)	(4)	(5)	(6)	(7)	
Conditions variable:	Area of district	Total population	Population density	Nighttime lights	NDVI	Urban share	Cropland share	
Crossed	−0.00954	−0.00428	−0.00674	−0.00514	−0.0325	−0.00675	−0.00590	
threshold	(0.0284)	(0.0292)	(0.0283)	(0.0284)	(0.0344)	(0.0282)	(0.0291)	
Conditions	−0.00477	0.0337***	0.0000107***	0.00162**	−0.00000670	0.130**	0.0228	
	(0.00234)	(0.00863)	(0.00000212)	(0.000426)	(0.00000433)	(0.0369)	(0.0218)	
Crossed	0.00265	−0.00757	−0.00000455	−0.000654	0.00000519	−0.0513	−0.00514	
threshold = 1 × Conditions	(0.00330)	(0.00391)	(0.00000504)	(0.000588)	(0.00000542)	(0.0626)	(0.0113)	
								
Observations	105140	105060	105080	105140	105140	105120	105120	

**Panel B**								
	(1)	(2)	(3)	(4)	(5)	(6)	(7)	(8)
Conditions variable:	Road dist. to cities	Dist. to coast	Dist. to waterway	Dist. to petroleum	Dist. to gold	Dist. to diamonds	Conflict fatalities	Princ. Comp.

Crossed	−0.00752	−0.00993	−0.00797	−0.00588	−0.00245	−0.00850	−0.00805	−0.00794
threshold	(0.0275)	(0.0292)	(0.0271)	(0.0282)	(0.0289)	(0.0308)	(0.0291)	(0.0289)
Conditions	−0.0000251**	−0.0000222*	−0.0000660	0.0000191	0.0000189	−0.0000267	−0.000677	
	(0.00000686)	(0.00000989)	(0.0000632)	(0.0000275)	(0.0000237)	(0.0000138)	(0.00394)	
Crossed	−0.00000190	0.00000433	−0.00000593	−0.00000871	−0.0000169	0.000000415	0.0188***	
threshold = 1 × Conditions	(0.00000396)	(0.0000163)	(0.000106)	(0.0000140)	(0.0000144)	(0.00000418)	(0.00382)	
PC 1								0.0143***
								(0.00242)
PC 2								0.00720
								(0.00491)
PC 3								−0.0135
								(0.00646)
Crossed								−0.00420
X PC1								(0.00376)
Crossed								−0.00252
X PC2								(0.00425)
Crossed								0.000104
X PC3								(0.00371)
								
Observations	104960	105140	105140	105140	105140	105140	103620	103500

Standard error clustered by country and year in parentheses. *** p<0.01, ** p<0.05, * p<0.1.

The threshold crossing appears to counteract these correlations slightly, but in general the coefficients on the interaction terms for nearly all variables are not statistically distinguishable from zero. The only meaningful statistical effect appears to be on the correlation with conflict fatalities. Crossing the LMIC threshold substantially increases the probability that an aid project will be located in a district that experienced higher conflict fatalities at baseline. This represents a significant extension of World Bank funding to districts that had experienced conflict. To be clear, we use our measures of conflict fatalities during the early 1990’s as a baseline measure to avoid endogeneity concerns; in some instances, these districts may still be experiencing conflict after the threshold crossing, while in other cases, these may be post-conflict settings.

While we observe strong correlations between our conditions and whether a district sees projects sited in its borders, we do not find such correlations with the funding amounts for these projects (Table 5). In fact, the only cross-sectional correlation appears to be with NDVI, as districts with higher mean greenness measures see smaller committed funds per capita. However, after crossing the LMIC threshold, funding amounts significantly increase for districts that have higher urban shares. There is a large but not significant increase in districts with higher NTL and those that are more densely populated as well, although these effects are too small and noisy to detect (Table 4). For example, the magnitudes of the coefficients imply that districts with one standard deviation more nighttime lights (SD  = 9.87) generally experience 1.6 percentage points higher probabilities of having any new aid projects (a 21.6% increase over the mean probability of 7.4%). After crossing the threshold, this drops by 0.6 percentage points to a 1.0 percentage points higher probability (interaction coefficient of -0.000654* SD of 9.87=-0.00645 drop). In other words, the interaction effect is only equivalent to approximately 40% of the cross-sectional correlation between need and aid, and thus not large enough to undo the strong correlation of aid locations with better need conditions.

**Table 5:** New funding per capita in district (US$ 000’s)

**Panel A**								
	(1)	(2)	(3)	(4)	(5)	(6)	(7)	
Conditions variable:	Area of district	Total population	Population density	Nighttime lights	NDVI	Urban share	Cropland share	
Crossed	−76.63	110.5	−304.3***	−2741.7	−9671.3	−1339.8	−4774.1	
threshold	(549.7)	(518.8)	(0.00127)	(1404.8)	(13963.9)	(1265.4)	(3638.1)	
Conditions	−805.2	−611.9	0.0668	274.5	−4.053	23373.5	−4515.0	
	(914.0)	(556.3)	(0.246)	(255.0)	(2.103)	(15285.4)	(4075.8)	
Crossed	588.2	200.4	1.613	625.7	2.115	54232.2*	11047.7	
threshold = 1 × Conditions	(762.8)	(896.1)	(1.665)	(449.0)	(2.941)	(24280.2)	(7437.8)	
								
Observations	105040	105040	105040	105040	105040	105040	105040	

**Panel B**								
	(1)	(2)	(3)	(4)	(5)	(6)	(7)	(8)
Conditions variable:	Road dist. to cities	Dist. to coast	Dist. to waterway	Dist. to petroleum	Dist. to gold	Dist. to diamonds	Conflict fatalities	Princ. Comp.

Crossed	2379.3**	3288.4	3653.2	2099.4	−2458.7	−614.9	−361.3	215.8
threshold	(645.6)	(2192.0)	(2153.8)	(1849.9)	(2360.4)	(1793.9)	(426.4)	(406.7)
Conditions	−2.067	−0.874	16.63	14.90	1.286	0.137	1037.8	
	(12.15)	(3.272)	(54.08)	(10.41)	(2.284)	(3.137)	(1511.6)	
Crossed	−6.191	−7.813	−80.46	−7.043	7.808	1.238	44324.8***	
threshold = 1 × Conditions	(13.53)	(8.165)	(78.88)	(12.22)	(7.836)	(4.574)	(900.8)	
PC 1								790.7
								(1437.4)
PC 2								2144.1
								(1193.3)
PC 3								−234.3
								(1057.7)
Crossed								4090.9*
X PC1								(1950.6)
Crossed								−810.5
X PC2								(2322.3)
Crossed								503.8
X PC3								(1031.8)
								
Observations	104900	105040	105040	105040	105040	105040	103580	103500

Standard error clustered by country and year in parentheses. *** p<0.01, ** p<0.05, * p<0.1.

The first principal component, which effectively combines a number of these variables, also exhibits a significant increase in project funding after threshold crossing. This indicates that while more urban settings may not be any more likely to see projects after threshold crossing, the funding they receive does appear to increase.

Importantly, we also observe large and significant increases in funding commitments for districts with higher conflict fatalities, consistent with our findings on whether any projects are sited in these districts. This is also consistent with our results on the number of project locations per district (See Table 14 in the Appendix). Taken together, the results on project siting and funding amounts indicate that the World Bank significantly increased levels of aid to conflict-affected locations after a country crossed the LMIC threshold (thereby confirming H1 for conflict conditions).

We also test our second hypothesis by using the natural log of committed funds as our outcome measure in the same specification. This allows us to test for efficient responses to tightening budgets after LMIC threshold crossing even in the case in which the marginal costs of delivering aid to districts are the same. In Table 6, we find largely similar patterns to those when considering outcomes in levels. More funds are initially provided to projects located in districts with greater population counts, population densities, NTL, and urban shares. Thus, we see large and significant correlations of aid funding with the first principal component. However, we do not see significant changes after the threshold crossing along most of these conditions and the first component. At the same time, we do see a large and significant increase in the log of committed funds provided to districts with higher conflict fatalities. Again, we can reject the null hypothesis for H2 for this conflict dimension, but not for our other measures. It appears that in terms of both the levels and ratios of aid provided, World Bank funding disproportionately flows to conflict-affected areas in response to tightening budgets.

**Table 6:** Ln(funding) in district (US$ 000’s).

**Panel A**								
	(1)	(2)	(3)	(4)	(5)	(6)	(7)	
Conditions variable:	Area of district	Total population	Population density	Nighttime lights	NDVI	Urban share	Cropland share	
Crossed	−0.0841	−0.0350	−0.0646	−0.0572	−0.336	−0.0673	−0.0582	
threshold	(0.243)	(0.251)	(0.243)	(0.242)	(0.322)	(0.241)	(0.258)	
Conditions	−0.0375	0.252***	0.0000906***	0.0147***	−0.0000691	1.202**	0.104	
	(0.0231)	(0.0568)	(0.0000189)	(0.00341)	(0.0000389)	(0.314)	(0.179)	
Crossed	0.0173	−0.0783**	−0.0000340	−0.00389	0.0000557	−0.289	−0.0384	
threshold = 1 × Conditions	(0.0291)	(0.0254)	(0.0000403)	(0.00505)	(0.0000552)	(0.552)	(0.112)	
								
Observations	105040	105040	105040	105040	105040	105040	105040	

**Panel B**								
	(1)	(2)	(3)	(4)	(5)	(6)	(7)	(8)
Conditions variable:	Road dist. to cities	Dist. to coast	Dist. to waterway	Dist. to petroleum	Dist. to gold	Dist. to diamonds	Conflict fatalities	Princ. Comp.

Crossed	−0.0566	−0.0765	−0.0665	−0.0595	−0.0306	−0.0795	−0.0762	−0.0754
threshold	(0.232)	(0.249)	(0.233)	(0.243)	(0.242)	(0.265)	(0.250)	(0.246)
Conditions	−0.000183**	−0.000176	−0.000685	0.000202	0.000203	−0.000188	−0.000147	
	(0.0000560)	(0.0000948)	(0.000643)	(0.000252)	(0.000203)	(0.000122)	(0.0464)	
Crossed	−0.0000539	0.00000259	−0.000212	−0.0000600	−0.000131	0.00000598	0.259***	
threshold = 1 × Conditions	(0.0000517)	(0.000159)	(0.00109)	(0.000137)	(0.000155)	(0.0000521)	(0.0491)	
PC 1								0.116***
								(0.0192)
PC 2								0.0623
								(0.0445)
PC 3								−0.0882
								(0.0533)
Crossed								−0.0233
X PC1								(0.0326)
Crossed								−0.0327
X PC2								(0.0425)
Crossed								0.0114
X PC3								(0.0342)
								
Observations	104900	105040	105040	105040	105040	105040	103580	103500

Standard error clustered by country and year in parentheses. *** p<0.01, ** p<0.05, * p<0.1.

## 6 Robustness checks

We consider the robustness of our estimates to the inclusion of additional controls for district-specific unobservables, differential time trends for each country, longer lags between threshold crossing and aid changes, and different specifications for both our estimation and our conditions variables.

In Table 7, we add district fixed effects to our baseline specification for project siting in a district, accounting for time-invariant district unobservables (beyond the country-wide unobservables accounted for by the country fixed effects in our baseline models). The interaction terms remain largely unaffected, as project siting only changes after LMIC threshold crossing based on the conflict fatalities conditions across districts.

**Table 7:** Any new projects in district, adding district fixed effects.

**Panel A**								
	(1)	(2)	(3)	(4)	(5)	(6)	(7)	
Conditions variable:	Area of district	Total population	Population density	Nighttime lights	NDVI	Urban share	Cropland share	
Crossed	−0.00979	−0.00678	−0.00666	−0.00621	−0.0185	−0.00767	−0.00325	
threshold	(0.0286)	(0.0291)	(0.0283)	(0.0283)	(0.0376)	(0.0282)	(0.0285)	
Crossed	0.00316	−0.00274	−0.00000481	−0.000426	0.00000220	−0.0192	−0.0110	
threshold = 1 × Conditions	(0.00348)	(0.00385)	(0.00000524)	(0.000522)	(0.00000556)	(0.0614)	(0.00924)	
								
Observations	105140	105060	105080	105140	105140	105120	105120	

**Panel B**								
	(1)	(2)	(3)	(4)	(5)	(6)	(7)	(8)
Conditions variable:	Road dist. to cities	Dist. to coast	Dist. to waterway	Dist. to petroleum	Dist. to gold	Dist. to diamonds	Conflict fatalities	Princ. Comp.

Crossed	−0.00655	−0.00976	−0.00840	−0.00726	−0.00660	−0.00874	−0.00811	−0.00795
threshold	(0.0273)	(0.0287)	(0.0271)	(0.0281)	(0.0291)	(0.0305)	(0.0291)	(0.0290)
Crossed	−0.00000465	0.00000390	0.00000406	−0.00000358	−0.00000474	0.000000763	0.0236***	
threshold = 1 × Conditions	(0.00000364)	(0.0000165)	(0.000113)	(0.0000146)	(0.0000225)	(0.00000370)	(0.00491)	
Crossed								−0.00259
X PC1								(0.00384)
Crossed								−0.00106
X PC2								(0.00400)
Crossed								0.00107
X PC3								(0.00445)
								
Observations	104960	105140	105140	105140	105140	105140	103620	103500

Standard error clustered by country and year in parentheses. *** p<0.01, ** p<0.05, * p<0.1.

It is also possible that the threshold crossing is temporally correlated with longer-term changes in countries’ aid flows and socioeconomic developments. Because our baseline specification includes only country fixed effects and common time fixed effects, it does not adjust for such longer-term changes in aid specific to a given country. If these longer-term changes are in fact correlated with the timing of the LMIC threshold crossing, this could be a challenge to our causal identification. To account for this, we thus add country-specific time trends as controls to our baseline specification, thereby accounting for smooth changes over the 20-year sample window that are specific to individual countries (beyond the sample-wide changes occurring in a given year that are accounted for by our year fixed effects). This is akin to estimating only the discontinuous changes in aid allocation following threshold crossing, a la Galiani et al. (2017). In Table 8, we show effects on project siting in a district with this specification. We again find significant interaction effects between threshold crossing and conflict fatalities. No other interaction terms exhibit effects significant at the 5% level.

**Table 8:** Any new project in district, adding country-specific trends.

**Panel A**								
	(1)	(2)	(3)	(4)	(5)	(6)	(7)	
Conditions variable:	Area of district	Total population	Population density	Nighttime lights	NDVI	Urban share	Cropland share	
Crossed	0.000268	0.00658	0.00203	0.00349	−0.0419*	0.00196	0.00762	
threshold	(0.0273)	(0.0285)	(0.0273)	(0.0277)	(0.0176)	(0.0273)	(0.0265)	
Conditions	−0.00408**	0.0349**	0.0000103***	0.00159***	−0.00000843	0.125**	0.0261	
	(0.00141)	(0.00996)	(0.00000190)	(0.000408)	(0.00000450)	(0.0349)	(0.0224)	
Crossed	0.00153	−0.0100	−0.00000305	−0.000562	0.00000930*	−0.0388	−0.0143	
threshold = 1 × Conditions	(0.00101)	(0.00672)	(0.00000361)	(0.000561)	(0.00000421)	(0.0544)	(0.0174)	
								
Observations	105140	105060	105080	105140	105140	105120	105120	

**Panel B**								
	(1)	(2)	(3)	(4)	(5)	(6)	(7)	(8)
Conditions variable:	Road dist. to cities	Dist. to coast	Dist. to waterway	Dist. to petroleum	Dist. to gold	Dist. to diamonds	Conflict fatalities	Princ. Comp.

Crossed	−0.00370	0.00192	0.00277	0.0175	0.0104	−0.0213	0.00183	0.00134
threshold	(0.0273)	(0.0311)	(0.0262)	(0.0285)	(0.0326)	(0.0359)	(0.0280)	(0.0274)
Conditions	−0.0000328***	−0.0000197*	−0.0000531	0.0000356	0.0000240	−0.0000411*	0.00311	
	(0.00000771)	(0.00000756)	(0.0000419)	(0.0000301)	(0.0000234)	(0.0000172)	(0.00381)	
Crossed	0.0000138*	−0.00000216	−0.0000410	−0.0000545*	−0.0000290	0.0000345	0.0111***	
threshold = 1 × Conditions	(0.00000568)	(0.0000109)	(0.0000558)	(0.0000253)	(0.0000176)	(0.0000168)	(0.00204)	
PC 1								0.0145***
								(0.00232)
PC 2								0.00815
								(0.00462)
PC 3								−0.0161*
								(0.00644)
Crossed								−0.00444
X PC1								(0.00239)
Crossed								−0.00540
X PC2								(0.00356)
Crossed								0.00697*
X PC3								(0.00322)
								
Observations	104960	105140	105140	105140	105140	105140	103620	103500

Standard error clustered by country and year in parentheses. *** p<0.01, ** p<0.05, * p<0.1.

As described in the Data section, we test for changes in aid allocations in the replenishment period following the LMIC threshold crossing. It is possible that these changes take time to materialize, so we may not observe such effects after longer lags. In Table 9, we use a three year lag in our *crossed* variable (reflecting changes in the next three-year replenishment period), as well as its interaction with our conditions variables. We continue to find evidence of shifts in project siting toward conflict-affected districts.

**Table 9:** Any new project in district, crossover lagged three years.

**Panel A**										
	(1)	(2)	(3)	(4)	(5)	(6)	(7)			
Conditions variable:	Area of district	Total population	Population density	Nighttime lights	NDVI	Urban share	Cropland share			
Crossed	−0.0206	−0.0203	−0.0215	−0.0206	−0.0124	−0.0219	−0.0182			
threshold (t-3)	(0.0295)	(0.0293)	(0.0276)	(0.0282)	(0.0296)	(0.0282)	(0.0303)			
Conditions	−0.00124	0.0315*	0.00000890***	0.00138**	−0.00000322	0.104**	0.0258			
	(0.00117)	(0.0125)	(0.00000205)	(0.000374)	(0.00000614)	(0.0304)	(0.0284)			
Crossed	−0.00318	−0.00385	−0.00000268	−0.000363	−0.00000209	−0.0140	−0.00968			
(t-3) X Conditions	(0.00162)	(0.00911)	(0.00000817)	(0.000558)	(0.00000946)	(0.0638)	(0.0322)			
										
Observations	89369	89301	89318	89369	89369	89352	89352			

**Panel B**										
	(1)	(2)	(3)	(4)	(5)	(6)	(7)	(8)		
Conditions variable:	Road dist. to cities	Dist. to coast	Dist. to waterway	Dist. to petroleum	Dist. to gold	Dist. to diamonds	Conflict fatalities	Princ. Comp.		

Crossed	−0.00286	−0.00375	−0.00302	−0.00343	−0.00174	−0.00210	−0.00313	−0.00347		
threshold	(0.0285)	(0.0284)	(0.0282)	(0.0287)	(0.0271)	(0.0277)	(0.0297)	(0.0294)		
Conditions	−0.0000177	−0.0000248***	−0.0000542	0.0000197	0.0000263	−0.0000137	−0.000596			
	(0.0000108)	(0.00000334)	(0.0000425)	(0.0000250)	(0.0000252)	(0.0000170)	(0.00673)			
Crossed	−0.0000183	0.00000707	−0.0000470	−0.00000823	−0.0000398	−0.0000295*	0.0258*			
(t-3) X Conditions	(0.0000194)	(0.00000982)	(0.000131)	(0.0000228)	(0.0000353)	(0.0000138)	(0.00975)			
PC 1								0.0128***		
								(0.00299)		
PC 2								0.00463		
								(0.00621)		
PC 3								−0.0120		
								(0.00938)		
Crossed								−0.00235		
(t-3) X PC1								(0.00413)		
Crossed								0.00369		
(t-3) X PC2								(0.00769)		
Crossed								−0.00622		
(t-3) X PC3								(0.0111)		
										
Observations	89216	89369	89369	89369	89369	89369	88077	87975		

Standard error clustered by country and year in parentheses. *** p<0.01, ** p<0.05, * p<0.1.

Finally, we consider whether these changes are driven by non-linearities and outliers among the small number of conflict-affected districts in our data. Recall that our measures are based on the mean conflict fatalities between 1990 and 1995 recorded in the UCDP dataset. In this data, only 9% of districts see any fatalities in this time period. We thus explore whether the effects on aid allocations are due to increasing funding to this subsample or to changes in allocations within this subsample. We find evidence of adjustments on both of these margins. In Table 10, we first show effects on project siting and committed funds per capita with our conditions variable now formulated as a dummy indicating any conflict fatalities in 1990–95. We continue to see differential changes on both outcomes toward districts with any conflict. We then limit our sample to only these conflict-affected districts and use the continuous measure of mean fatalities on the right hand side. We find that even among this sample, there are significant changes in aid siting and funding based on conflict conditions, with worse off districts seeing aid projects more frequently and with greater funding. Finally, we confirm that these effects are not due to leverage exerted on linear estimation due to the presence of disproportionately high conflict districts. We use the natural log of conflict fatalities as our measure of conditions in the full sample, again finding a similar pattern of effects. Taken together, these tests confirm that tightening aid budgets cause World Bank aid projects to be increasingly devoted to conflict-affected zones.

**Table 10:** Conflict.

	Any projects in district			Committed funds in district				
	(1)	(2)	(3)	(4)	(5)	(6)		
	Conflict as dummy	Continuous measure in conflict-affected sample	Ln(conflict)	New aid commitments per capita (000’s)	New aid commitments per capita (000’s)	New aid commitments per capita (000’s)		
Crossed threshold	−0.0116	0.0384	0.0829	−1567.1	15251.2	79283.2***		
	(0.0302)	(0.0270)	(0.0454)	(809.7)	(10435.1)	(475.3)		
1(Conflict	0.00391			−630.8				
fatalities)	(0.00925)			(1959.4)				
Crossed threshold = 1	0.0427			19867.6*				
× 1(Conflict fatalities)	(0.0256)			(8302.7)				
Conflict fatalities		−0.00691**			701.9			
		(0.00202)			(1166.0)			
Crossed threshold = 1		0.0111*			40611.0***			
× Conflict fatalities		(0.00474)			(4771.8)			
Ln(conflict			0.00138			1411.2		
fatalities)			(0.00431)			(1635.7)		
Crossed threshold = 1			0.0201			17506.8***		
× Ln(conflict fatalities)			(0.0110)			(1187.4)		
								
Observations	103620	9780	103620	103580	9780	103580		

Standard error clustered by country and year in parentheses. *** p<0.01, ** p<0.05, * p<0.1.

## 7 Health sector

Donor targeting in the health sector is of particular importance because a large array of interventions have been shown to cost effectively reduce disease burdens and mortality rates, but existing evidence suggests highly imperfect targeting of these interventions to the populations with highest needs (Kotsadam et al., 2018). This is particularly concerning both because of the scale of the resources devoted to health sector aid (the WB alone committed more than $100 B to the sector during our study period) and because, as we describe in our Conceptual Framework, much of the literature on health aid suggests impacts may be greater in higher-need locations.

We therefore focus our analysis on the subsample of health aid projects, assessing their allocation relative to standardized measures of population health. There are many such potential measures, but few of these are consistently available for our pre-1995 period in which our geocoded IDA-IBRD project sample begins. We thus limit our sample to Bolivia, Cameroon, Ghana, India, Indonesia, and Nigeria. Helpfully, several of these are large, diverse countries, providing substantial variation in health conditions prior to their crossing the LMIC threshold.

We utilize the DHS surveys to construct baseline measures of population health, focusing on child mortality and morbidity rates, as these are known to be particularly sensitive to health conditions. We calculate child mortality rates from the DHS birth histories for the pre-1995 DHS surveys available. DHS sample sizes make the estimates representative at the first administrative unit (typically region or state) rather than district level. Our unit of analysis is thus the region-year. Because these regions vary substantially in population and size, we weight our analysis by population.

We narrow our sectoral coverage to only projects with a health-related theme identified in the WB database. The IDA-IBRD database contains 272 health projects in the six countries that were approved between 1995 and 2014, entailing $4,193,205,237 in committed funds.

In Table 11, we estimate effects on project siting and funding outcomes at the region level. We begin by estimating our baseline specification including country and year fixed effects to account for time-invariant unobservables at the country level and sample-wide annual movements in aid siting. In column 2, we add country-specific trends that account for smooth changes taking place in each country over the 1995–2014 sample period. In column 3, we further add region-specific fixed effects that adjust for time-invariant unobservables at this subnational scale, and column 4 adds country-year fixed effects. Throughout, we do not find a significant interaction between the initial mortality rate in each region and the threshold crossing.

**Table 11:** Health Aid and Child Mortality.

	Any projects in region					Committed funds in district					
	(1)	(2)	(3)	(4)	(5)	(6)	(7)	(8)	(9)	(10)	
	Baseline	+ Country Trends	Region FEs	+ CountryYear FEs	Mortality Bins	Mortality Bins	Unweighted	New Commitments	Ln(Commitments)	Mortality Bins	
Crossed threshold	0.05719	0.1788	0.1727		0.07006	0.9399***	0.1143**			2.7260*	
	(0.1963)	(0.1722)	(0.1601)		(0.1287)	(0.06830)	(0.04932)			(1.4939)	
Initial Child Mortality X Crossed threshold	0.00003066	-0.001165	-0.001109	-0.0007955				−6343.8	-0.003780		
	(0.0009040)	(0.0007359)	(0.0007099)	(0.0006624)				(31717.6)	(0.008189)		
Initial Child Mortality	0.0006691	0.0009411									
	(0.0006282)	(0.0006144)									
Crossed threshold = 1 × Child mortality bin minimum = 50					-0.09952***	−0.1031***	-0.08885*			−1.4198*	
					(0.02073)	(0.03346)	(0.04662)			(0.7082)	
Crossed threshold = 1 × Child mortality bin minimum = 100					−0.1349***	−0.1378	−0.1069*			−2.5393*	
					(0.04382)	(0.08784)	(0.05507)			(1.4132)	
Crossed threshold = 1 × Child mortality bin minimum = 150					−0.1557***	−0.1632**	−0.1327**			−3.1386***	
					(0.01485)	(0.06290)	(0.05221)			(1.0533)	
Crossed threshold = 1 × Child mortality bin minimum = 200					−0.1103	−0.1499**	−0.1201**			−2.5970	
					(0.07005)	(0.06821)	(0.05000)			(1.6060)	
											
Country FE	Y	Y	–	–	–		–	Y	Y	Y	
Year FE	Y	Y	Y	Y	Y	Y	Y	Y	Y	Y	
Country Trends	N	Y	Y	N	N	N	N	N	N	N	
Region FE	N	N	Y	Y	Y	Y	Y	N	N	N	
Country X Year FE	N	N	N	Y	N	Y	Y	N	N	N	
P-value Crossed X Mortality Bin 50 = Crossed X Mortality Bin 150					0.00009424	0.3543	0.2185			0.1126	

Standard errors clustered by region and year in parentheses.

It is possible that our interaction of threshold crossing with a linear term in initial child mortality masks responses that are vary non-linearly across the initial mortality distribution. We explore this by decomposing initial mortality into five bins (0–49, 50–99, 100–149, 150–199, and ⩾200 per 1,000 births). We show these results in columns 5–7. Once we adjust for country-year-specific unobservables in columns 6 and 7, we find that crossing the threshold increased the likelihood that regions with the lowest initial mortality receive health aid projects. Regions with higher mortality rates receive aid projects at differentially lower rates after the threshold crossing. The differential changes decline across these bins, albeit non-monotonically at the top end of the mortality distribution.

We statistically test whether the changes post-threshold for regions in the 50–99 mortality bin and those in the 150–199 bin are equal. These differential changes are statistically distinguishable when we include year and country fixed effects and smooth country trends (col 5). After adjusting for country-year-specific unobservables (col 6), these effects are no longer statistically distinguishable. Column 7 confirms that this finding is not due to the use of population weights by repeating the specification in Column 6 without weighting the analysis by population.

We find quite similar results when we consider as our outcome variable the amount of committed funds in the region (cols 8–10). Again, we find changes post-crossing that do not vary linearly in the initial child mortality distribution, with coefficients that suggest lower allocations for regions in the 50–199 mortality rate bins than those with the lowest mortality. However, these differential changes post-crossing are not statistically distinguishable.

We also consider additional measures of child health beyond mortality rates. We use the same pre-1995 DHS survey waves to calculate the prevalence of diarrhea and fever in children under 5 in each region. In Table 12, we estimate the effects on project siting and committed funding, with region and year fixed effects, as well as either country-specific trends or country-year fixed effects. The interaction between threshold crossing and initial health conditions is not significantly different from zero at the 95% confidence level for any of the specifications or health measures. We estimate a small, positive coefficient on the interaction between crossover and diarrhea prevalence in column 2 (significant at the 90% confidence level), but this slight effect does not survive when changing outcome or health measures.

**Table 12:** Health Aid and Child Morbidity.

	Diarrhea Prevalence				Fever Prevalence				
	(1)	(2)	(3)	(4)	(5)	(6)	(7)	(8)	
	Any Project in Region	Any Project in Region	Committed funds in region	Committed funds in region	Any Project in Region	Any Project in Region	Committed funds in region	Committed funds in region	
Crossed threshold	−0.06904		0.004341		−0.07719		−0.004190		
	(0.1615)		(0.1244)		(0.1670)		(0.1256)		
Initial Diarrhea Prev. X Crossed threshold	0.01657	0.02885*	−0.05328	−0.1026					
	(0.02180)	(0.01391)	(0.03883)	(0.06203)					
Initial Fever Prev. X Crossed threshold					0.01330	0.01876	−0.02435	-0.02476	
					(0.01587)	(0.01085)	(0.01929)	(0.01809)	
									
Region FE	Y	Y	Y	Y	Y	Y	Y	Y	
Year FE	Y	Y	Y	Y	Y	Y	Y	Y	
Country Trends	Y	–	Y	–	Y	–	Y	–	
Country X Year FE	N	Y	N	Y	N	Y	N	Y	

Standard errors clustered by region and year in parentheses.

Taken together, these results confirm that health aid is no better targeted towards health needs after the threshold crossing. Our findings do not support either of the hypotheses from our conceptual framework, indicating that WB health aid does not efficiently respond to budget shocks through geographic reallocation.

## 8 Offsetting differences in costs?

As we detail in our conceptual framework, it is possible that differences in marginal costs across locations of varying need may dampen the response to tightening budgets. In particular, if areas of high need are also those where aid activities are most expensive to implement, an efficient response to a shrinking budget may see only small increases in the share of funding provided in these areas. Our null results on changes in aid based on districts’ population, economic activity, and geophysical characteristics may thus reflect efficient responses if costs differ substantially across these characteristics. Theory is ambiguous about whether such cost differences are likely to occur: while frictions can give rise to price variations in locally sourced inputs and labor, donors and implementers source many inputs from international or selected domestic suppliers. We therefore explore empirically whether project costs differ substantially across regions of varying conditions, particularly those along which we do not detect changes in aid allocations after LMIC threshold crossing. We find little evidence of any such correlation, with or without extensive fixed effects adjusting for unobservables.

We overcome several key data constraints presented by the WB data. First, the WB provides only the total costs of each project, spread over all its locations, inclusive of management and oversight costs. We therefore conduct the analysis at the project level, averaging the need measures over each project’s locations to create our explanatory variable of interest. As our dependent variable, we use the total funds committed by the project, as well as the total funds divided by number of locations and, separately, total funds divided by population in all serviced locations.

Our second constraint is that although we would like to know average rather than total costs, the WB does not consistently provide quantities of inputs used by each project. To overcome this, we use project evaluation data, particularly the project’s implementation success rating, as a control variable. In doing so, we assume that projects with greater implementation success likely used fewer inputs to provide greater outputs (or used similar quantities of inputs to provide greater outputs, or both). These project implementation ratings are generally provided by the WB Task Team Leaders and validated or replaced by ratings from the WB Independent Evaluation Group (IEG) in a subset of cases.

To study the empirical relationship with as much statistical power as possible, we use the full sample of World Bank projects in the geocoded IDA-IBRD datasbase. We address the potential correlation of project characteristics with unobserved confounds by adopting country, year, and sector fixed effects. We thus estimate the following specification:

$${\mathrm{Costs}}_{\mathrm{pcst}}=\beta_{0}+\beta_{1}{\mathrm{Need}}_{\mathrm{pc}}+\beta_{2}{\mathrm{OverallRating}}_{\mathrm{pcst}}+\beta_{3}{\mathrm{ImpRating}}_{\mathrm{pcst}}+D_{c}+D_{t}+D_{s}+{∊}_{\mathrm{pcst}}$$

where ${\mathrm{Costs}}_{\mathrm{pcst}}$ represents the costs for project *p* in country *c* focused on sector *s* begun in year $t,{\mathrm{Need}}_{\mathrm{pc}}$ is the mean of the need measures across all locations serviced by the project, *OverallRating* and *ImpRating* are the project’s overall and implementation evaluation scores, and $D_{c},D_{t}$ and $D_{s}$ are country, year, and sector fixed effects. We cluster our standard errors by country and year.

Our findings indicate that project costs are not correlated with population density, NTL, or NDVI in the locations they serve, as shown in the Table 13 above. This is true irrespective of whether we use total project costs, project costs per location, or project costs per capita as our outcome measure. We observe very weak positive correlations with population density and NTL that are not statistically different from zero. Greenness, as measured by NDVI, is associated with slightly lower project costs, but again the correlation is quite weak and not statistically different from zero. Taken together, these results confirm that the non-response of WB project sites and funding to tightening budgets does not efficiently account for differences in need across locations.

**Table 13:** Costs analysis.

DV = Total amounts per location						
Luminosity	0.0692	0.0998				
	(0.126)	(0.131)				
Population			2.35e-05	2.57e-05		
			(2.20e-05)	(2.26e-05)		
NDVI					−0.00451	−0.00480
					(0.00325)	(0.00335)
Constant	−27.48*	−34.20	−28.29*	−34.98	−21.05	−27.53
	(15.04)	(24.65)	(15.16)	(24.64)	(13.93)	(22.99)
FEs	Y	Y	Y	Y	Y	Y
Project Rating Controls	N	Y	N	Y	N	Y
N	34,411	33,941	34,411	33,941	34,411	33,941
Observations	0.150	0.162	0.150	0.162	0.150	0.162

Note: Standard errors in parentheses. *** p<0.01, ** p<0.05, ∗ p<0.1.

## 9 Conclusions

Our results suggest that, at least among a particularly large and influential multilateral donor, project siting and funding does not appear to efficiently respond to tightening budget constraints, except in the case of conflict-affected districts. One explanation of our results is that policymakers use a different information set about conditions. They may not have had district-level data on the conditions we consider at their disposal, and possibly may use their own experiences or other, local sources of information on conditions. We cannot rule out the case in which the policymakers’ information set better reflects conditions than do our measures. Nonetheless, we consider this an unlikely situation, as we have incorporated a wide array of measures collected from both satellite- and survey-based observations, and compiled this into particularly powerful principal components.

A second explanation of our null findings is that policymakers and local citizens have a different social welfare function than that posited in our Conceptual Framework. For example, policymakers and citizens may strongly prefer allocations that shift only slowly over time because they may allow for less variability and more reliable longer-term planning for local governments and households alike. Indeed, while there is some evidence from its Country Assistance Strategies that the World Bank has sought to shift in priorities in the wake of partner country transitions out of IDA eligibility and changing overall aid budgets, there is also evidence of continuity. For example, the 2006 Country Assistance Strategy in Honduras emphasized that, “[i]nstitutionalization and continuity are critical for achieving development objectives” in advocating for a continuation of past funding priorities (World Bank, 2006, 15). At the same time, it is also likely that citizens prefer at least proportional allocations (and potentially more progressive ones), but the cross-sectional data show allocations that are *positively* correlated with wealth. To justify the efficiency of our results, policymakers and citizens would need to have preferences that are quite uncommon. It is therefore unlikely that our results show efficient targeting outcomes. However, we cannot rule out the possibility that observed allocations are in line with an alternative set of preferences. Still, while the preferences we assume here may not reflect the Bank’s true preferences, they are a useful baseline for comparison in the sense that they comport not only with the Bank’s stated goals but also how the international community and interested observers believe aid ought to be allocated.

Who actually controls decisions over the siting and funding of aid projects within a country is an important question, one from which we abstract. Experience suggests that foreign donors, national governments, and implementing agency staff all play a role in these decisions, with the locus of control shifting as the geographic units become smaller. For example, donors and national governments may have strong preferences over allocations across regions but leave decisions over specific villages within districts to implementers. It is nonetheless important for actors across this spectrum to have at their disposal sufficiently rich geographic data on conditions to support efficiency aims.

We focus on an important but understudied margin on which aid allocation decisions are made. The growing availability of geocoded data on donor-supported activities from AidData and other sources provides rich opportunities for further research along these dimensions. In particular, integrating the political economy considerations and projected impacts derived from evaluation work into realistic models of geographic allocation would offer a particularly powerful direction for future work. Similarly, the growth of funding from non-Western donors provides opportunities to explore whether these donors are more responsive to changing budget constraints (or to changes in funding from Western donors). It is important to note that this work was facilitated by the use of GeoQuery, allowing the researchers to integrate subnational data. GeoQuery’s primary source of vector data is geoBoundaries, an online, open license resource of geographic boundaries, which are easily accessible for public usage and provides administrative zone information for nearly all countries at the ADM0, ADM1, and ADM2 levels (Goodman et al., 2019). To the authors knowledge, the geoBoundaries dataset is the only global administrative database that is provisioned with a full quality assurance procedure (Runfola et al., 2020). While geoBoundaries builds on numerous efforts within the geographic community to establish high quality geographic data by preferencing the most precise information available, the boundary files are not perfect. Further improvements in geoBoundaries to expand higher levels of granularity in administrative hierarchies, increase precision in boundary files, and expand boundary data into a time series format are critical to continuing to address concerns in data representation, processing and geovisualization of administrative divisions (Runfola et al., 2020).

Our findings speak to numerous related studies. First, our analysis reinforces the existing findings by Briggs (2021) and others by extending the set of indicators considered and applying an identification strategy from the cross-national aid allocation literature. Across more than a dozen indicators of local need, we find very little evidence that changing a country’s IDA-eligibility status leads the World Bank to shift its funding priorities in accordance with need. Second, our approach parallels and complements findings from the country-level aid allocation literature. Just as Collier and Paul (2001) and Collier and Paul (2002) show that the cross-country targeting of foreign aid departs from what an optimal efficient allocation would demand, our findings suggest that donors allocate aid sub-optimally within recipient countries. This has important implications for the aid effectiveness debate. Indeed, if aid was allocated efficiently within countries, the misallocation of aid (from a poverty-reduction standpoint) at the cross-country level may be less worrisome. However, our results and the findings of other related studies suggest that this relatively optimistic perspective may be unwarranted. It is important to note that although optimal targeting does not guarantee optimal implementation (e.g., Devahive et al., 2015) and a lack of optimal targeting does not imply that aid cannot promote local development progress (e.g., Dreher et al., 2021), it is a basic prerequisite for increasing the overall efficiency and effectiveness of aid.

Third, we show the potential promise of employing identification strategies from the country-level allocation literature to answer questions about aid allocation and effectiveness within countries. Although we find mostly null results, this approach should help increase confidence that our (null) findings are not explained by unobserved confounders. At the same time, our findings suggest that scholars interested in studying the local effects of aid on conflict may consider examining changing IDA-eligibility status in combination with recent conflict fatalities as they try to untangle the effectiveness of aid in reducing the impacts of conflict. Finally, our findings speak to and potentially complicate debates over explanations for the observed rich-region bias in aid allocation. Whereas other studies have identified the potential for variation in implementation costs to explain the positive correlation between subnational wealth and aid allocation (e.g., Maiden and Brockway, 2018, Briggs, 2021), our study empirically investigates that possibility using data on project costs from World Bank project documents. We find little evidence that underlying costs are driving the observed differences in allocation across regions of at varying levels of need. Future work should explore further how variation in the burdens of providing development assistance shape allocation decisions within countries.

One caveat is that, due to limited data, we cannot systematically account for the activities of other donors at the subnational level in modeling the World Bank’s allocation decisions. It is possible that the decision to site projects in certain regions is shaped by strategic considerations about how doing so will complement or reinforce other development efforts. This is a question that is worth investigating as availability of geocoded data on aid project locations extends to a broader set of donors. However, existing research suggests that donors often fail to coordinate their allocations at the cross-national level (Aldasoro, Nunnenkamp, & Thiele, 2010), and recent work suggests that this is largely true of donor behavior at the subnational level as well. For example, in their analysis of the case of Malawi, (Nunnenkamp, Sotirova, & Thiele, 847 (2016)) “do not find compelling evidence for increased aid specialization after the Paris Declaration, and the regional division of labour among donors may even have deteriorated.” Other working papers from Öhler (2013) and Nunnenkamp et al. (2016) report similar findings in the cases of Cambodia and Uganda, respectively.

Still, to consider the possibility that donors are strategically coordinating their aid in ways that would affect our core results, we examine the case of Nigeria, which is the only of our cases that crosses the IDA threshold in our sample for which we have geocoded data from multiple donors using AidData’s Nigeria AIMS Geocoded Research Release, Version 1.3.2 (AidData, 2016). The majority of geocoded locations are coded at the ADM1 level in this data set (58%), so we aggregate project location codings up to the ADM1 level. For each ADM1-level region in each year, we measure (1) the number of new projects that were started by the World Bank and (2) the number of projects started by any other donor or combination of donors in the data set. Table 16 in the Appendix reports the results of this analysis, which shows that the allocation of other donors’ projects is not significantly correlated with the World Bank’s subnational allocation of projects. We find that the estimates are all near zero and change sign depending on the model specification. This provides at least some limited evidence that the World Bank’s behavior is not systematically driven by the behavior of other donors. Finally, we note that, even if there were strategic interactions between the Bank’s and other donors’ siting and allocation decisions, these interactions would have to be very strong and pronounced in order to overcome the disproportionate siting of Bank projects (which represents a large share of overall aid) in less poor areas. In other words, the Bank would have to expect that other donors would allocate almost all of their own funding toward the most poor areas in order to justify directing its own funding disproportionately to less poor areas.

Do our results based on countries’ transition to lower-middle income status readily translate to countries that remain low-income? Our findings complement the work by Briggs (2021) and others whose samples do include many low-income countries. By studying LMIC transitions, we also examine specific periods of opportunity for major changes in aid strategies and allocations. Finding little evidence of such changes in these opportune moments suggests targeting is not likely improving rapidly outside these periods. Our null results on efficient targeting also likely extend to other multilateral and bilateral donors. In fact, if even the WB – which is frequently seen as a particularly technocratic multilateral institution with documented targets and extensive data at hand – appears not to target its aid efficiently at subnational scales, other donors may be even less likely to do so.

## CRediT authorship contribution statement

**Ariel BenYishay:** Conceptualization, Methodology, Formal analysis, Writing - original draft. **Matthew DiLorenzo:** Conceptualization, Formal analysis, Writing - review & editing. **Carrie Dolan:** Conceptualization, Methodology, Writing - review & editing.

## Declaration of Competing Interest

The authors declare that they have no known competing financial interests or personal relationships that could have appeared to influence the work reported in this paper.

## Figures and Tables

**Table 14:** Number of new locations in district.

**Panel A**									
	(1)	(2)	(3)	(4)	(5)	(6)	(7)		
Conditions variable:	Area of district	Total population	Population density	Nighttime lights	NDVI	Urban share	Cropland share		
Crossed	−12.58	−12.77	−12.36	−12.76	−11.78	−12.50	−14.20		
threshold	(12.75)	(12.89)	(12.58)	(12.29)	(7.805)	(12.13)	(12.42)		
Conditions	−0.941	−0.904	−0.000363	0.0640	−0.00343	10.99	1.153		
	(0.947)	(0.660)	(0.000299)	(0.0764)	(0.00195)	(12.34)	(4.567)		
Crossed	1.017	1.346	0.000878	0.145	−0.0000630	14.72	4.707		
threshold = 1 × Conditions	(1.032)	(1.152)	(0.000723)	(0.124)	(0.00185)	(22.06)	(2.942)		
									
Observations	104960	104960	104960	104960	104960	104960	104960		

**Panel B**									
	(1)	(2)	(3)	(4)	(5)	(6)	(7)	(8)	
Conditions variable:	Road dist. to cities	Dist. to coast	Dist. to waterway	Dist. to petroleum	Dist. to gold	Dist. to diamonds	Conflict fatalities	Princ. Comp.	

Crossed	−12.96	−13.00	−13.97	−11.42	−17.40	−16.98	−12.82	−12.17	
threshold	(14.67)	(16.00)	(16.38)	(12.81)	(17.59)	(17.11)	(12.67)	(12.58)	
Conditions	−0.00701	−0.00461	−0.0389	0.0150	−0.00437	−0.00129	0.0849		
	(0.00910)	(0.00594)	(0.0790)	(0.00938)	(0.00773)	(0.00289)	(0.316)		
Crossed	0.00249	0.00236	0.0444	−0.00246	0.0156	0.00732	43.16***		
threshold = 1 × Conditions	(0.00678)	(0.00985)	(0.101)	(0.00527)	(0.0156)	(0.00729)	(2.537)		
PC 1								1.072	
								(1.748)	
PC 2								0.0557	
								(1.726)	
PC 3								−1.513	
								(1.744)	
Crossed								0.675	
X PC1								(1.777)	
Crossed								1.846	
X PC2								(3.456)	
Crossed								−0.0563	
X PC3								(0.951)	
									
Observations	104900	104960	104960	104960	104960	104960	103500	103500	

Standard error clustered by country and year in parentheses. *** p<0.01, ** p<0.05, * p<0.1.

**Table 15:** New Infrastructure Project in District?

**Panel A**								
	(1)	(2)	(3)	(4)	(5)	(6)	(7)	
Conditions variable:	Area of district	Total population	Population density	Nighttime lights	NDVI	Urban share	Cropland share	
Crossed	−0.00144	0.00314	−0.0000857	−0.000131	0.0163	−0.000383	0.000605	
threshold	(0.00611)	(0.00734)	(0.00595)	(0.00574)	(0.0113)	(0.00580)	(0.00731)	
Conditions	−0.000914	0.0101***	0.00000346	0.000421	0.00000165	0.0413	0.00751	
	(0.000750)	(0.00158)	(0.00000176)	(0.000216)	(0.000000845)	(0.0202)	(0.00661)	
Crossed	−0.0000294	−0.00882***	−0.00000422	−0.000281	−0.00000381	−0.0372	−0.00455	
threshold = 1 × Conditions	(0.00119)	(0.00223)	(0.00000245)	(0.000290)	(0.00000203)	(0.0266)	(0.00955)	
								
Observations	105140	105060	105080	105140	105140	105120	105120	

**Panel B**								
	(1)	(2)	(3)	(4)	(5)	(6)	(7)	(8)
Conditions variable:	Road dist. to cities	Dist. to coast	Dist. to waterway	Dist. to petroleum	Dist. to gold	Dist. to diamonds	Conflict fatalities	Princ. Comp.

Crossed	−0.00305	−0.00633	−0.00554	−0.00126	−0.00976	0.00198	−0.000737	−0.000535
threshold	(0.00664)	(0.00662)	(0.00666)	(0.00846)	(0.00774)	(0.00977)	(0.00626)	(0.00582)
Conditions	−0.00000929*	−0.0000102**	−0.0000720*	0.00000707	−0.00000850	−0.00000391	0.000836	
	(0.00000409)	(0.00000340)	(0.0000301)	(0.0000110)	(0.0000127)	(0.00000692)	(0.00147)	
Crossed	0.00000462	0.0000124	0.0000956*	-0.000000720	0.0000242	−0.00000514	0.00199	
threshold = 1 × Conditions	(0.00000598)	(0.00000620)	(0.0000455)	(0.0000117)	(0.0000141)	(0.00000780)	(0.00335)	
PC 1								0.00459**
								(0.00138)
PC 2								−0.00117
								(0.00126)
PC 3								−0.00192
								(0.00213)
Crossed								−0.00339
X PC1								(0.00211)
Crossed								0.00292
X PC2								(0.00189)
Crossed								−0.00155
X PC3								(0.00268)
								
Observations	104960	105140	105140	105140	105140	105140	103620	103500

Standard error clustered by country and year in parentheses. *** p<0.01, ** p<0.05, * p<0.1.

**Table 16:** Donor coordination in Nigeria.

	*Dependent variable:*				
	DV = N. World Bank projects				
	(1)	(2)	(3)		
N. other donor projects	−0.007	0.014	−0.005		
	(0.019)	(0.011)	(0.021)		
Constant	0.356***	−0.014	−1.098**		
	(0.058)	(0.434)	(0.532)		
					
Year dummies:	No	Yes	Yes		
Region dummies:	No	No	Yes		
Observations	289	289	270		
R^2^	0.0004	0.695	0.825		
Adjusted R^2^	−0.003	0.672	0.783		
Residual Std. Error	0.758 (df = 287)	0.434 (df = 267)	0.358 (df = 217)		
F Statistic	0.126 (df = 1; 287)	29.040*** (df = 21; 267)	19.710*** (df = 52; 217)		

*Note:*	∗p<0.1; **p<0.05; ***p<0.01				

## Footnotes

1 However, there may be important differences across different types of donors. For instance, Dipendra (2020) shows that bilateral and multilateral aid targets those most in need better than aid from (international) nongovernmental organizations in Nepal.

2 An alternative approach might entail using this non-homothetic utility function to first show that the ratio of aid to different regions is not constant with respect to the total aid budget, and then explore how the slope of the resulting income expansion path varies with respect to conditions.

3 Corner solutions are possible in which all aid is allocated to the region with the highest need, but in practice in our data, there are no countries in which all aid is provided only to the highest need location.

4 One possible direction for future research would be to extend the model to incorporate the possibility of the Samaritan’s Dilemma, with donors’ response to need creating perverse incentives for recipient governments to exert less effort (a la Hagen (2006)). Such an adaptation would have to account for the empirical facts discussed in Section 2 of greater aid funding flowing to subnational areas with better conditions rather than worse, undercutting recipients’ expectations that donors will respond to worsening needs.

5 The AidData geocoding methodology does not specify the value of funding provided to each location, but does approximate these values with even splits of the total committed funding divided by the number of locations identified.

6 During our study period (based on the availability of geo-referenced aid data), there are also 12 countries which moved from LMIC to LIC status (although two of these are Nigeria and Cameroon, both of which are in our sample, and first transitioned from LIC to LMIC status before fluctuating and then finally transitioning to LMIC status more stably). A large segment of the other countries are former Soviet republics (including Belarus, Bulgaria, Kazakhstan, Lithuania, and Ukraine), and funding changes for these countries were heavily colored by revamping of state enterprises and market reforms. More generally, the overall funding changes in these LMIC to LIC transitions for countries are much less consistent than those in LIC to LMIC direction, making it more difficult to draw generalizable inferences about the response to aid budget changes.

7 The loadings on the second and third principal components do not lend themselves to intuitive interpretation. This is not uncommon in PCA analysis, particularly since the first component is derived to maximize its variance and subsequent components must be orthogonal to it, thus exhibiting less variance and requiring greater nuance in interpretation. In our case, the second component most heavily weights NDVI (negatively) and distance to the coast (positively), suggesting it reflects interior districts with less dense vegetation patterns. The third component most heavily weights cropland share (negatively), indicating it may reflect less arable conditions.

8 We include the conditions variables one-by-one in the model rather than jointly because multicollinearity would likely compromise our ability to detect any effects. The fact that we find few significant interaction effects in our main results means the interaction effects in a joint model would almost certainly be insignificant.
